# Neoadjuvant PD-(L)1 blockade plus platinum-based chemotherapy for potentially resectable oncogene-positive non-small cell lung cancer

**DOI:** 10.1186/s12957-024-03434-1

**Published:** 2024-06-18

**Authors:** Xuchen Zhang, Hefeng Zhang, Feng Hou, Tao Fang, Chuantao Zhang, Huiyun Wang, Shanai Song, Hongwei Lan, Yongjie Wang, Helei Hou

**Affiliations:** 1 Precision Medicine Center of Oncology, The Affiliated Hospital of Qingdao University, No. 59 Haier Road, Qingdao, Shandong 266035 China; 2 Department of Thoracic Surgery, The Affiliated Hospital of Qingdao University, No. 59 Haier Road, Qingdao, Shandong 266035 China; 3 Department of Pathology, The Affiliated Hospital of Qingdao University, Qingdao University, No.59 Haier Road, Qingdao, 266035 China; 4 Department of Oncology, Shengli Oilfield Central Hospital, No.31 Jinan Road, Dongying, Shandong 257099 China; 5 Department of Oncology, The Affiliated Hospital of Qingdao University, No. 7 Jiaxing Road, Qingdao, Shandong 266031 China

**Keywords:** Neoadjuvant therapy, Immune checkpoint blockade, Oncogene-positive non-small cell lung cancer, Retrospective study, PD-L1

## Abstract

**Background:**

Whether programmed cell death-1/ligand-1 (PD-1/PD-L1) blockade-based neoadjuvant treatment may benefit locally advanced oncogene-mutant non–small cell lung cancer (NSCLC) patients remains controversial. This retrospective study was designed to observe the efficacy and safety of neoadjuvant PD-1/PD-L1 blockade plus chemotherapy versus chemotherapy and corresponding tyrosine kinase inhibitors (TKIs) in patients with resectable oncogene-positive NSCLC.

**Methods:**

Patients with potential resectable NSCLC harbouring oncogene alterations who had received neoadjuvant treatment were retrospectively recruited, and an oncogene-negative cohort of patients who received neoadjuvant PD-(L)1 blockade-based neoadjuvant treatment was reviewed for comparison during the same period. The primary aim was to observe the treatment efficacy and event-free survival (EFS) of these agents. Safety profile, molecular target, and immunologic factor data, including PD-L1 expression and tumour mutational burden (TMB), were also obtained.

**Results:**

A total of 46 patients were recruited. Thirty-one of them harboured oncogene alterations, including *EGFR*, *KRAS*, *ERBB2*, *ROS1*, *MET*, *RET*, *ALK*, and *FGFR3* alterations. Among the oncogene-positive patients, 18 patients received neoadjuvant PD-(L)1 blockade immunotherapy plus chemotherapy (oncogene-positive IO group), 13 patients were treated with neoadjuvant chemotherapy and/or corresponding TKIs or TKIs alone (oncogene-positive chemo/TKIs group), and the other 15 patients were oncogene negative and received neoadjuvant PD-(L)1 blockade plus chemotherapy (oncogene-negative IO group). The pathological complete response (pCR) and major pathological response (MPR) rates were 22.2% (4 of 18) and 44.4% (8 of 18) in the oncogene-positive IO group, 0% (*P* = 0.120) and 23.1% (3 of 13) (*P* = 0.276) in the oncogene-positive chemo/TKIs group, and 46.7% (7 of 15) (*P* = 0.163) and 80.0% (12 of 15) (*P* = 0.072) in the oncogene-negative IO group, respectively. By the last follow-up, the median EFS time had not reached in the oncogene-positive IO group, and was 29.5 months in the oncogene-positive chemo/TKIs group and 38.4 months in the oncogene-negative IO group.

**Conclusion:**

Compared with chemotherapy/TKIs treatment, neoadjuvant treatment with PD-(L)1 blockade plus platinum-based chemotherapy was associated with higher pCR/MPR rates in patients with partially resectable oncogene-mutant NSCLC, while the pCR/MPR rates were lower than their oncogene-negative counterparts treated with PD-(L)1 blockade-based treatment. Specifically, oncogene alteration types and other predictors of response to immunotherapy should be taken into account in clinical practice.

**Supplementary Information:**

The online version contains supplementary material available at 10.1186/s12957-024-03434-1.

## Introduction

Lung cancer remains a severe medical burden with high morbidity and mortality worldwide, with an estimated 2.2 million cases and 1.8 million deaths in 2020 [1]. In China, non-small cell lung cancer (NSCLC) is the predominant pathological type and includes oncogene alterations such as *EGFR* and *ALK*, in a large proportion of patients [2].

The widespread developments of immune checkpoint blockades (ICBs), notably antibodies targeting programmed cell death receptor-1/ligand-1 (PD-1/PD-L1), have greatly broadened the treatment options for solid tumours including NSCLC. Nevertheless, in advanced NSCLCs with positive oncogene mutations such as *EGFR* and *ALK* alterations, anti-PD-1/PD-L1 treatment exhibited inferior efficacy than corresponding tyrosine-kinase inhibitors (TKIs) according to the IMMUNOTARGET study [3–5], and the inferior response might be caused by lower PD-L1 expressions, lower tumour mutational burden (TMB), and fewer CD8^+^ tumour infiltrating lymphocytes and neoantigens in oncogene-positive NSCLCs [6]. Inspiringly, the results of the IMpower-150 trial and the ORIENT-31 trial indicated that the combined treatment regimen of immunotherapy, chemotherapy and anti-angiogenesis agents might be effective in *EGFR*-mutant advanced NSCLC patients [7–9], while the potential mechanisms warranted further explorations.

For decades, neoadjuvant chemotherapy followed by surgical resection has remained the standard treatment strategy for potential resectable NSCLC [10]. Preoperative PD-1 blockade plus chemotherapy has shown superior clinical outcomes over chemotherapy alone in neoadjuvant settings [11–13], and this combinational approach has been approved in potential resectable oncogene-negative NSCLCs. Nevertheless, whether neoadjuvant PD-1 blockade plus chemotherapy could be applied in resectable NSCLCs harbouring oncogene alterations such as *EGFR* and *ALK* remains dubious. Recently, several studies have been focused on exploring the efficacy of neoadjuvant PD-1/PD-L1 blockade-based therapy in oncogene-positive NSCLCs. In LCMC3 trial, none of the *EGFR/ALK-*altered stage IB to IIIA NSCLC patients had achieved major pathological response (MPR) after PD-L1 blockade atezolizumab monotherapy. Encouragingly, in a recent study observing the efficacy of neoadjuvant PD-L1 blockade atezolizumab plus chemotherapy, two of four *EGFR*-mutant patients achieved pathological complete response (pCR), indicating NSCLCs harbouring oncogene alterations might also benefit from this combinational treatment regimen [14]. Another two studies also presented potentially promising results of neoadjuvant PD-1/PD-L1 blockade-based treatment in NSCLCs harbouring various driver gene alteration types [15, 16], while the MPR and pCR rates varied with different studies.

Moreover, neither the interaction between PD-L1 expression and oncogene alterations in NSCLC nor the predictive value of PD-L1 expression and TMB in neoadjuvant immunotherapy are clear. At present, the challenge is how to reliably identify patients who could benefit from neoadjuvant anti-PD-(L)1-based immunotherapy plus chemotherapy. Herein, we designed this retrospective study to observe the efficacy and safety of neoadjuvant PD-(L)1 blockade plus platinum-based chemotherapy versus neoadjuvant chemotherapy with or without corresponding TKIs in patients with resectable oncogene-positive NSCLCs; in addition, we compared the efficacy of neoadjuvant PD-(L)1 blockade-based treatment between oncogene-positive and oncogene-negative NSCLC cohorts.

## Methods

After having been reviewed and approved by the Institutional Review Board of the Affiliated Hospital of Qingdao University (Qingdao, China, trial registration number: QYFYWZLL27970), we retrospectively assembled the clinical information of treatment-naïve, baseline clinical stage IIA to IIIB, potentially resectable, oncogene-positive NSCLC patients from September 2019 to July 2023 at the Affiliated Hospital of Qingdao University; additionally, we recruited oncogene-negative patients who received neoadjuvant immunotherapy for comparison during the same period. The major inclusion criteria included the following: (a) patients diagnosed with locally advanced stage IIA to IIIB and potential resectable NSCLC both radiographically and pathologically; (b) patients harbouring sensible oncogenic alterations and oncogene-negative patients who received neoadjuvant PD-(L)1 blockade-based treatment; (c) patients with no previous systematic antitumour treatment; (d) patients with an Eastern Cooperative Oncology Group performance status (ECOG PS) score of 0 or 1 (on a 5-point scale, within which a higher score is equal to more severe disabilities); and (e) patients with adequate hepatic, renal, pulmonary, and haematopoietic functions. The major exclusion criteria were as follows: (a) had malignancies other than lung cancer; (b) had distant metastatic lesions; (c) had undergone more than six cycles of neoadjuvant treatment or had multiple lines of preoperative treatment; and (d) had inadequate vital organ function or autoimmune disease.

### Treatment

The enrolled patients received two to six cycles of neoadjuvant treatment, and the treatment regimens were PD-(L)1 blockade immunotherapy plus chemotherapy, chemotherapy plus corresponding TKIs, or TKIs alone. The radiographic tumour response to neoadjuvant treatment was assessed after every two cycles of treatment, and surgery was planned to be performed approximately four weeks after the last dose of neoadjuvant treatment according to multidisciplinary discussions. PD-(L)1 blockade involved the use of one of the following regimens: pembrolizumab (200 mg iv drip, every three weeks), camrelizumab (200 mg iv drip, every three weeks), tislelizumab (200 mg iv drip, every three weeks), sintilimab (200 mg iv drip, every three weeks), toripalimab (240 mg iv drip, every three weeks), or durvalumab (1000 mg iv drip, every three weeks). The chemotherapy regimen was standardized platinum-based dual drug chemotherapy.

### Assessments

The primary tumour at baseline and tumour response to neoadjuvant treatment were evaluated according to the Response Evaluation Criteria in Solid Tumours version 1.1 [17] using contrast-enhanced computed tomography (CT). Moreover, contrast-enhanced CT was performed approximately one month after surgery and subsequently every three months until recurrence or metastasis occurred or two years after surgery was reached.

Biopsies of tumour tissue were collected both at the time of diagnosis and during surgery, and surgical samples of primary tumours from the lung and regional lymph nodes were obtained according to the criteria of the American Joint Committee on Cancer (eighth edition). To evaluate the residual viable tumour cells and assess primary tumours, routine haematoxylin and eosin staining was used [18]. An absence of residual viable tumour cells was considered to indicate pCR [19], and if the proportion of residual viable tumour cells was no more than 10%, the pathological outcome was considered an MPR. Next-generation sequencing was performed for all the enrolled patients to obtain information on genomic alterations. Moreover, the expression of programmed death ligand-1 (PD-L1) was evaluated by immunohistochemical staining using the anti-PD-L1 antibody 22C3 (Dako, Glostrup, Denmark).

### Outcome evaluation

The outcomes of interest were pathological response, radiographic response (complete response, CR; partial response, PR; stable disease, SD; progressive disease, PD), EFS (the time from diagnosis to any one of the following three events: inability to proceed with surgery due to disease progression, local or distant relapsed disease, or death by any reason), and treatment-related adverse events (TRAEs) monitored according to the National Cancer Institute Common Terminology Criteria for Adverse Events version 5.0 [20].

## Results

### Patients and treatment

**Fig. 1:**
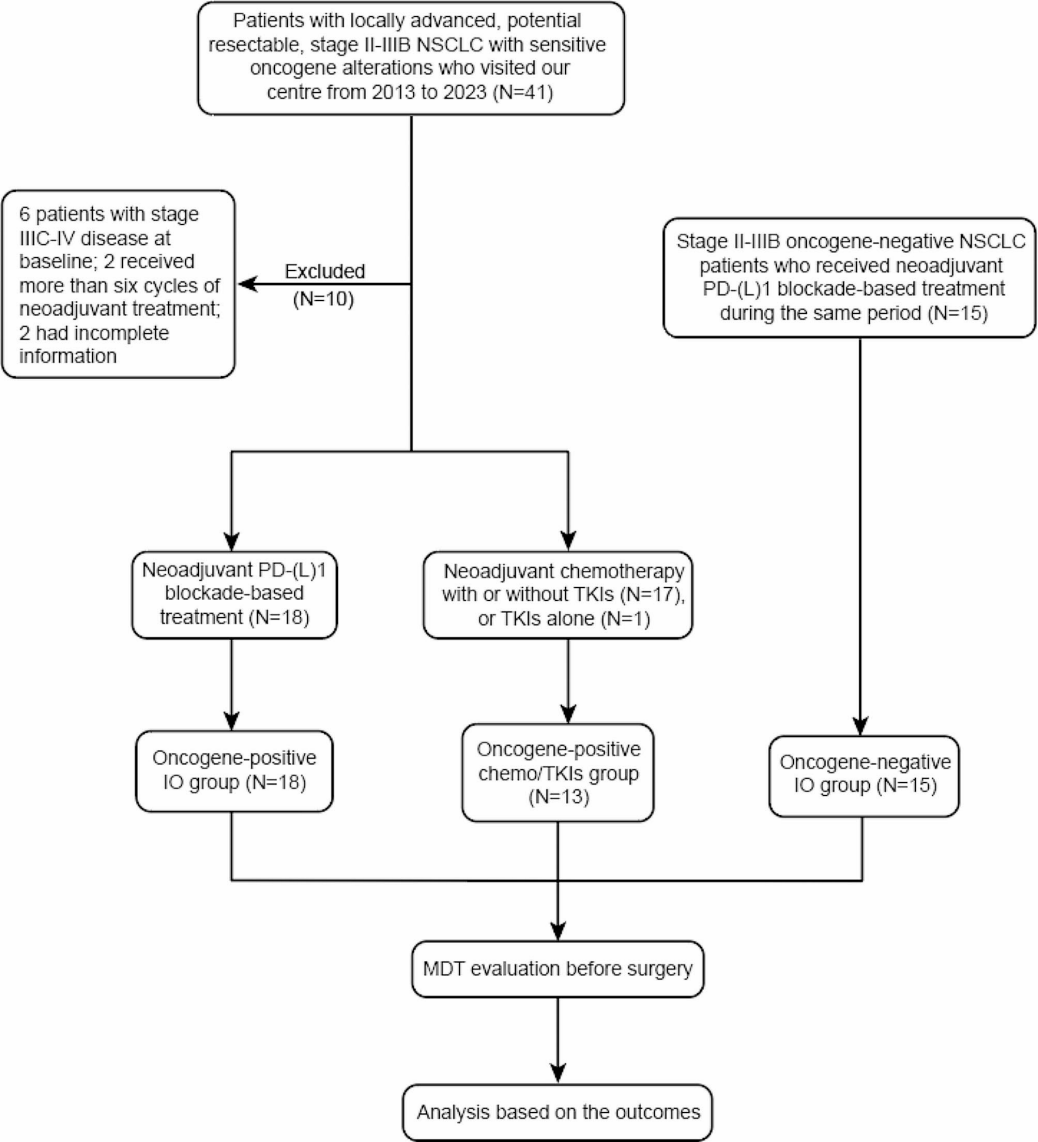
Flow chart of this study

A total of 46 patients were included in this study after strict screening (Fig. 1). Among the included patients, 31 harboured oncogene alterations—13 with *EGFR* alterations, seven with *KRAS* mutations, three with *ERBB2* alterations, two with alterations in *ROS1*, *MET*, and *RET*, and one with alterations in *ALK* and *FGFR3*. Among the oncogene-positive patients, 18 patients received neoadjuvant PD-(L)1 blockade plus chemotherapy (oncogene-positive IO group), 13 patients were treated with neoadjuvant chemotherapy and/or corresponding TKIs or TKIs alone (oncogene-positive chemo/TKIs group), and the other 15 patients were oncogene negative and received neoadjuvant PD-(L)1 blockade plus chemotherapy (oncogene-negative IO group). The PD-(L)1 blockade regimens used in each group were as follows: In the oncogene-positive IO group, there were seven patients treated with tislelizumab, four with pembrolizumab, three with camrelizumab, two with toripalimab, one with sintilimab, and one with one cycle of toripalimab plus three cycles of tislelizumab. In the oncogene-negative IO group, six patients were treated with toripalimab, three were treated with tislelizumab, two were treated with pembrolizumab, two were treated with camrelizumab, one was treated with sintilimab, and one was treated with durvalumab. In the oncogene-positive chemo/TKI group, seven patients were treated with chemotherapy alone, five patients with chemotherapy plus corresponding TKIs, and one patient with TKI monotherapy. The chemotherapy regimens used in these three groups were standardized platinum-based dual drug regimens (platinum-pemetrexed regimen, PP; or platinum-paclitaxel regimen, TP). The details of the treatment regimens used are listed in Table S1.

According to the eighth edition of the TNM classification for lung cancer, IASLC [21], all the patients were staged IIA to IIIB; 22.2% (4 of 18) of the patients in the oncogene-positive IO group, 23.1% (3 of 13) in the oncogene-positive chemo/TKI group, and 20.0% (3 of 15) in the oncogene-negative IO group had T_4_ disease; and 61.1% (11 of 18), 69.2% (9 of 13), and 66.7% (10 of 15) of the patients, respectively, had N_2_ disease. A total of 77.8% (14 of 18), 92.3% (12 of 13), and 13.3% (2 of 15) of patients had lung adenocarcinoma; one patient (5.6%) harbouring the *FGFR-TACC3* fusion in the oncogene-positive IO group was defined as having not otherwise specified NSCLC (NOS-NSCLC); and the other patient had squamous cell carcinoma.

**Table 1:** Demographics and baseline characteristics

Characteristics	Oncogene-positive IO group (*N* = 18)	Oncogene-positive chemo/TKIs group (*N* = 13)	Oncogene-negative IO group (*N* = 15)	*P*-value
Age (years)				0.122
Mean (Standard deviation)	62.5 (8.42)	56.8 (8.38)	61.1 (4.58)	
Gender, *n* (%)				0.0007
Male	11 (61.1)	3 (23.1)	14 (93.3)	
Female	7 (38.9)	10 (76.9)	1 (6.7)	
Pathological type, *n* (%)				< 0.0001
Adenocarcinoma	14 (77.8)	12 (92.3)	2 (13.3)	
Squamous cell carcinoma	3 (16.6)	1 (7.7)	13 (86.7)	
NSCLC-NOS	1 (5.6)	0	0	
Maximum tumour size, *n* (%)				0.114
1 cm < size ≤ 3 cm	4 (22.2)	7 (53.8)	2 (13.3)	
3 cm < size ≤ 5 cm	9 (50.0)	3 (23.1)	7 (46.7)	
5 cm < size ≤ 7 cm	5 (27.8)	2 (15.4)	3 (20.0)	
size ≥ 7 cm	0	1 (7.7)	3 (20.0)	
Baseline T staging, *n* (%)				0.581
T_1_	1 (5.6)	3 (23.1)	1 (6.7)	
T_2_	7 (38.9)	3 (23.1)	8 (53.3)	
T_3_	6 (33.3)	4 (30.7)	3 (20.0)	
T_4_	4 (22.2)	3 (23.1)	3 (20.0)	
Baseline N staging, *n* (%)				0.894
N_0_	3 (16.6)	3 (23.1)	3 (20.0)	
N_1_	3 (16.6)	1 (7.7)	2 (13.3)	
N_2_	11 (61.1)	9 (69.2)	10 (66.7)	
N_x_	1 (5.6)	0	0	
Baseline TNM, *n* (%)				0.666
II_A_	1 (5.6)	1 (7.7)	2 (13.3)	
II_B_	3 (16.7)	3 (23.1)	0	
III_A_	9 (50.0)	6 (46.1)	9 (60.0)	
III_B_	5 (27.7)	3 (23.1)	4 (26.7)	
Genetic alterations, *n* (%)				0.276
*EGFR/KRAS* alterations	10 (55.6)	10 (76.9)	-	
Other mutations	8 (44.4)	3 (23.1)	-	
Preoperative PD-L1 TPS, *n* (%)				0.214
<1%	4 (22.2)	6 (46.1)	3 (20.0)	
1 -50%	6 (33.3)	2 (15.4)	2 (13.3)	
≥50%	6 (33.3)	1 (7.7)	6 (40.0)	
Unknown	2 (11.1)	4 (30.8)	4 (26.7)	

**Fig. 2:**
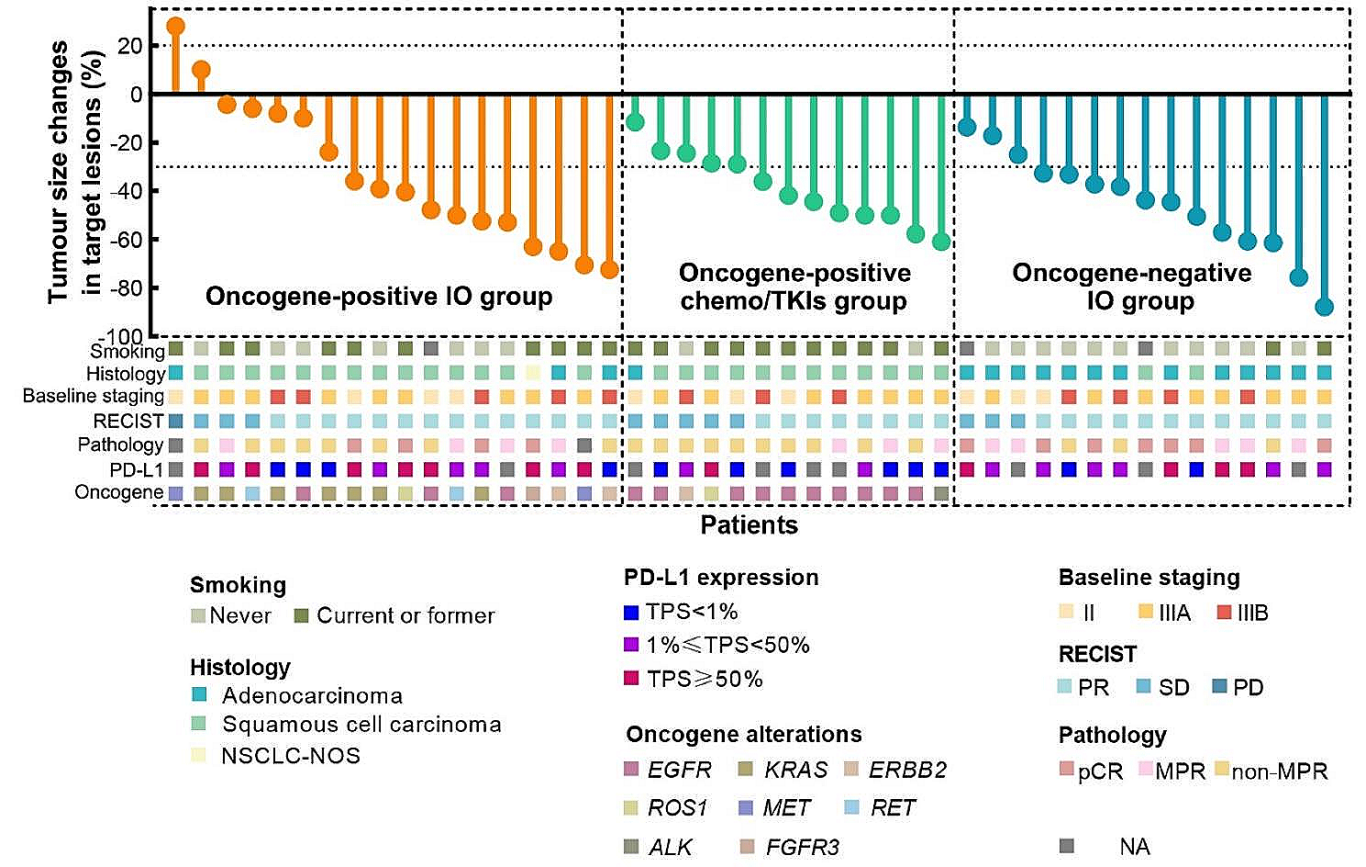
Baseline characteristics, oncogene alteration features, and tumour response in target lesions after neoadjuvant treatment. NSCLC-NOS, not otherwise specified non-small cell lung cancer; PR, partial response; SD, stable disease; PD, progressive disease; pCR, pathological complete response; MPR, major pathological response

Regarding the immunological factor data, 66.7% (12 of 18) of the patients in the oncogene-positive IO group, 23.1% (3 of 13) in the oncogene-positive chemo/TKI group, and 60.0% (9 of 15) in the oncogene-negative IO group had PD-L1 positivity (TPS ≥ 1%). Additionally, 22.2% (4 of 18) of patients in the oncogene-positive IO group and 15.4% (2 of 13) of patients in the oncogene-negative IO group had high TMB (≥ 10 muts/Mb). The baseline demographic data, oncogenic alteration characteristics, and immunological factor data are listed in Table 1 and S1 and Fig. 2.

### Clinical activity and tumour response

After the last cycle of neoadjuvant treatment, 12 of 18 (66.7%) patients in the oncogene-positive IO group, 8 of 13 (61.5%) patients in the oncogene-positive chemo/TKIs group, and 12 of 15 (80.0%) patients in the oncogene-negative IO group, respectively, reached PR, with ORRs of 66.7% (95% CI, 0.412–0.857), 61.5% (95%CI, 0.323–0.849), and 80.0% (95%CI, 0.514–0.947), respectively; 5 of 18 (27.8%), 5 of 13 (38.5%), and 3 of 15 (20.0%) patients, respectively, maintained SD, while one patient (5.6%) harbouring *MET* amplification in oncogene-positive IO group had disease progression after neoadjuvant treatment.

Sixteen of the 18 (88.9%) patients in the oncogene-positive IO group and all the patients in the oncogene-positive chemo/TKIs group and the oncogene-negative IO group underwent R0 surgical resection with lobectomy and lymphadenectomy. In the oncogene-positive IO group, one patient harbouring the MET exon 14 skipping mutation achieved a PR after neoadjuvant treatment and did not want to undergo surgery, and the patient with *MET*-amplified disease progression did not undergo surgery after multidisciplinary discussions.

Among the patients who had undergone surgical resection and had available surgical tumour tissue samples, pCR was achieved in 4 of 18 (22.2%, 95% CI 0.074–0.481) patients in the oncogene-positive IO group and 7 of 15 (46.7%, 95% CI 0.223–0.726) patients in the oncogene-negative IO group, while none of the patients in the oncogene-positive chemo/TKI group achieved pCR. Regarding MPR, 8 of 18 (44.4%, 95% CI 0.224–0.687) patients in the oncogene-positive IO group, 3 of 13 (23.1%, 95% CI 0.062–0.540) patients in the oncogene-positive chemo/TKI group, and 12 of 15 (80.0%, 95% CI 0.514–0.947) patients in the oncogene-negative IO group achieved MPR. In oncogene-positive patients, even though no statistically significant differences in pCR were detected between the IO group and the chemo/TKIs group, there was still a potential trend towards a higher pCR rate (4 of 18, 22.2%, versus 0 of 13, *P* = 0.120). (Table 2 A)

In the oncogene-positive IO group, the four patients who achieved pCR harboured the* ROS1*-*SDC4* fusion with high PD-L1 expression (TPS 99%) and high TMB (20.16 muts/Mb), the *KRAS G12A * fusion with high TMB (13.98 muts/Mb) and PD-L1 positivity (TPS 15%), the *KRAS G12C* fusion with high PD-L1 expression (TPS 75%), and the *FGFR3-TACC3* fusion with PD-L1 TPS 20%. The four patients harbouring the *EGFR exon 19* deletion, *ERBB2* amplification, *KRAS G12V*, and *RET-KIF5B* fusion had achieved MPR but not pCR. The details of the pathological response are shown in Table 2B.

**Table 2:** Tumour responses and surgical outcomes

**(A) Radiographical and pathological tumour response**							
	**vOncogene-positive IO group (** ***N*** ** = 18)**	**Oncogene-positive chemo/TKIs group (** ***N*** ** = 13)**	***P-*** **value** ^†^	**Oncogene-negative IO group (** ***N*** ** = 15)**		***P-*** **value** ^‡^	
Radiographic response, *n* (%)			0.601			0.539	
PR	12 (66.7)	8 (61.5)			12 (80.0)		
SD	5 (27.8)	5 (38.5)			3 (20.0)		
PD	1 (5.5)	0			0		
ORR	66.7%	61.5%			80.0%		
Pathological response*, *n* (%)							
pCR	4 (22.2)	0	0.120		7 (46.7)	0.163	
MPR	8 (44.4)	3 (23.1)	0.276		12 (80.0)	0.072	
**(B) Surgical outcomes**							
	**Oncogene-positive IO group (** ***N*** ** = 16)** ^#^	**Oncogene-positive chemo/TKIs group (** ***N*** ** = 13)**	***P-*** **value** ^†^		**Oncogene-negative IO group (** ***N*** ** = 15)**	***P-*** **value** ^‡^	
Type of resection, *n* (%)			0.460			0.091	
Lobectomy	12 (75.0)	10 (76.9)			6 (40.0)		
Bilobectomy	4 (25.0)	2 (15.4)			7 (46.7)		
Pneumonectomy	0	1 (7.7)			2 (13.3)		
Postoperative T staging, *n* (%)			0.207			0.396	
ypT_0_	4 (25.0)	0			7 (46.6)		
ypT_is_/T_1_	8 (50.0)	8 (61.5)			6 (40.0)		
ypT_2_	3 (18.8)	4 (30.8)			1 (6.7)		
ypT_3_	0	1 (7.7)			1 (6.7)		
ypT_4_	1 (6.2)	0			0		
Postoperative *N* staging, *n* (%)			0.222			0.994	
ypN_0_	12 (75.0)	6 (46.1)			11 (73.4)		
ypN_1_	2 (12.5)	2 (15.4)			2 (13.3)		
ypN_2_	2 (12.5)	5 (38.5)			2 (13.3)		

^#^P14 and P15 did not undergo surgery

^†^ The comparisons between oncogene-positive IO group and oncogene-positive chemo/TKIs group

^‡^ The comparisons between oncogene-positive IO group and oncogene-negative IO group

### Survival outcomes

**Fig. 3:**
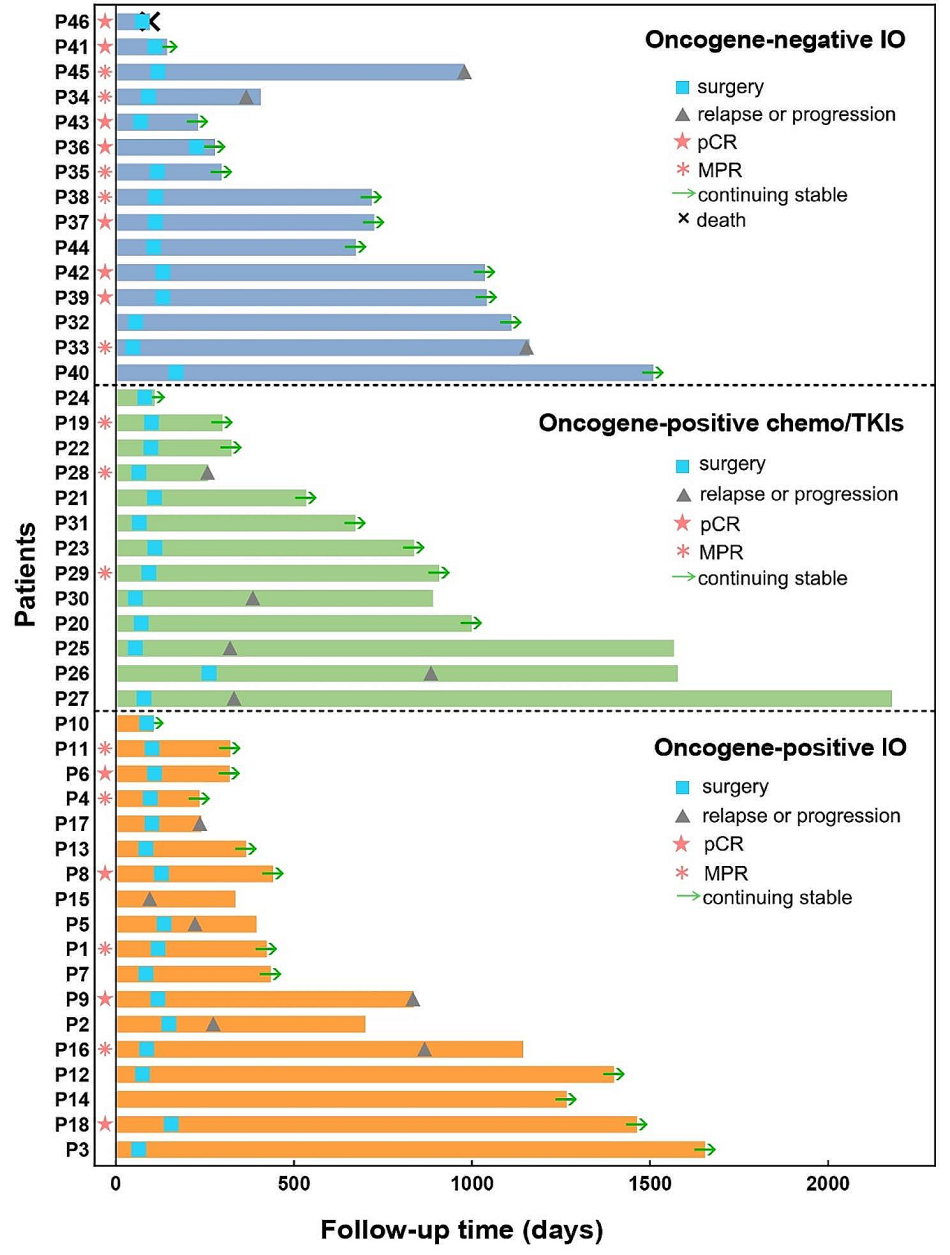
Swimmer plot of the treatment process and follow-up of the patients from the time of diagnosis

**Fig. 4:**
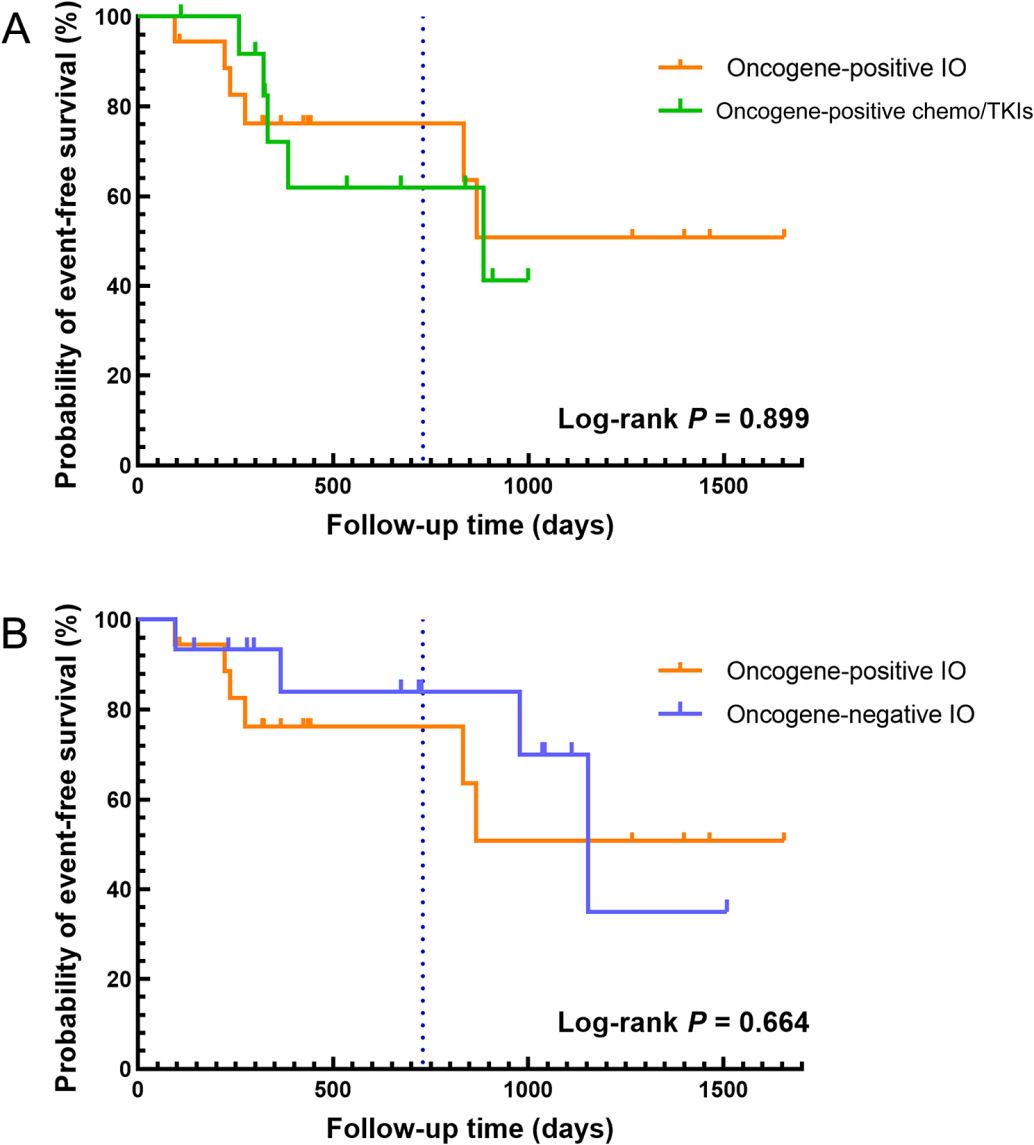
Kaplan-Meier event-free survival curves. **(A)** Oncogene-positive IO group versus oncogene-positive chemo/TKIs group (*P* = 0.899). **(B)** Oncogene-positive IO group versus oncogene-negative IO group (*P* = 0.664). The 2-year estimated EFS rates were 77.8% in the oncogene-positive IO group, 65.5% in the oncogene-positive chemo/TKIs group, and 80.8% in the oncogene-negative IO group

By the last follow-up on May 9, 2024, the median follow-up time was 14.1 months in the oncogene-positive IO group, 27.5 months in the oncogene-positive chemo/TKIs group, and 23.6 months in the oncogene-negative IO group. The median EFS time was 29.5 months in the oncogene-positive chemo/TKIs group, and 38.4 months in the oncogene-negative IO group, while had not been reached in the oncogene-positive IO group. The 2-year estimated EFS rates were 77.8% in the oncogene-positive IO group, 65.5% in the oncogene-positive chemo/TKIs group, and 80.8% in the oncogene-negative IO group, as shown in Fig. 3 and Fig. 4.

In the oncogene-positive IO group, a total of six patients reached the EFS endpoint, including one *MET*-amplified patient (P15) who did not undergo surgery, two patients harbouring *RET-KIF5B* with postoperative ilium bone metastasis (P16) and mediastinal lymph node metastasis (P17), one *EGFR L858R*-mutant patient (P2) with mediastinal lymph node metastasis, one *ERBB2*-amplified patient (P5) with liver metastasis, and one *KRAS G12C*-mutant patient with brain metastasis (P9). There were five patients (P25, 26, 27, 28, 30) in the oncogene-positive chemo/TKI group and four patients (P33, 34, 45, 46) in the oncogene-negative IO group who reached the EFS endpoint.

Notably, three patients in the oncogene-positive IO group were followed up for more than three years and were still alive without disease recurrence; these patients harboured *EGFRexon 18* alterations (P3), *MET exon 14* skipping mutations (P14), and the *ROS1-SDC4* fusion (P18), as shown in Fig. 3.

### Safety and feasibility

Regarding the safety profile, TRAEs of any grade during the neoadjuvant treatment period were observed in 10 of 18 (55.6%) patients in the oncogene-positive IO group, 11 of 13 (84.6%) in the oncogene-positive chemo/TKIs group, and 5 of 15 (33.3%) in the oncogene-negative IO group. Generally, the combined regimen of PD-1 blockade plus platinum-based chemotherapy in resectable oncogene-positive NSCLC patients was well tolerated, and the incidence of TRAEs was not significantly different from that in the oncogene-positive chemotherapy/TKI cohort (*P* = 0.129) or oncogene-negative immunotherapy cohort (*P* = 0.296).

Most TRAEs were grade 1 to 2 and were manageable, among which a decreased neutrophil count and increased alanine transaminase (ALT) and aspartate transaminase (AST) levels were the most common. However, in the oncogene-positive IO group, two patients who suffered from grade 3 immune-related hepatitis (P2) and grade 3 reactive cutaneous capillary hyperplasia (P15) were observed; these two conditions should be taken into consideration in clinical practice. No treatment-related deaths occurred. The details of the neoadjuvant TRAEs are presented in Table 3.

**Table 3:** Neoadjuvant treatment-related adverse events

TRAEs (*n*, %)	Oncogene-positive IO group (*N* = 18)		Oncogene-positive chemo/TKIs group (*N* = 13)			Oncogene-negative IO group (*N* = 15)		
	Grade 1–2	Grade 3–4	Grade 1–2	Grade 3–4	*P-*value^†^	Grade 1–2	Grade 3–4	*P-*value^‡^
Any grade	10 (55.6)		11 (84.6)		0.129	5 (33.3)		0.296
Grade 3–4	2 (11.1)		3 (23.1)		0.625	2 (13.3)		> 0.999
Decreased neutrophil count	3 (16.7)	0	4 (30.8)	0		1 (6.7)	2 (13.3)	
Anemia	1 (5.6)	0	3 (23.1)	0		0	0	
Decreased platelet count	0	0	0	1 (7.7)		2 (13.3)	0	
Increased bilirubin level	0	0	1 (7.7)	0		0	0	
Increased ALT level	6 (33.3)	0	5 (38.5)	0		3 (20.0)	0	
Increased AST level	6 (33.3)	0	1 (7.7)	0		2 (13.3)	0	
Diarrhoeal	0	0	0	2 (15.4)		0	0	
Rash	1 (5.6)	0	0	0		0	0	
Reactive cutaneous capillary hyperplasia	0	1 (5.6)	0	0		0	0	
Immune-related hepatitis	0	1 (5.6)	0	0		0	0	

^†^ The comparisons between oncogene-positive IO group and oncogene-positive chemo/TKIs group

^‡^ The comparisons between oncogene-positive IO group and oncogene-negative IO group

## Discussion

In locally advanced NSCLCs, neoadjuvant treatment is designed to transform the unresectable lesions into resectable ones and, further, to improve the surgical outcomes and long-term prognosis [22]. The phase 3 CheckMate-816 trial first demonstrated the superior pathological response outcomes of neoadjuvant PD-1 blockade nivolumab plus chemotherapy over chemotherapy alone, with an MPR rate of 36.9% and a pCR rate of 8.9% in *EGFR/ALK*-negative NSCLCs [23]; PD-1 blockade plus chemotherapy has become the milestone of neoadjuvant treatment for oncogene-negative locally advanced NSCLCs based on these results. Nevertheless, most of these studies precluded participants harbouring sensitive oncogene mutations including *EGFR/ALK*, and whether this combined regimen could benefit locally advanced oncogene-mutant NSCLC patients remains poorly explored. Currently, whether neoadjuvant immunotherapy plus chemotherapy would actually benefit locally advanced NSCLC patients harbouring driven alterations is controversial. In this study, we explored the efficacy and safety of neoadjuvant PD-1 blockade plus platinum-based chemotherapy versus chemotherapy/TKIs in locally advanced oncogene-mutant NSCLCs. We observed encouraging pathological response outcomes as well as a manageable safety profile in the patients harbouring *ROS1-SDC4* fusion and *FGFR3-TACC3* fusion regardless of PD-L1 expression levels and TMB status. In contrast, this combinational regimen showed satisfactory outcomes neither in the patient harbouring *EGFR L858R* point mutation with high TMB nor in the *MET*-amplified patient with low TMB. Additionally, the clinical outcomes varied among the patients harbouring *ERBB2* amplification, *KRAS* alterations, and *RET-KIF5B* fusion. Our finding suggests that neoadjuvant PD-(L)1 blockade plus immunotherapy might be explored in NSCLC patients harbouring a specific oncogene alteration type, which warrants further verification in clinical trials with larger cohorts. These results also highlighted the vital position of genetic testing in personalized precision treatment in oncogene-positive NSCLCs.

At present, prospective studies focusing on neoadjuvant use of PD-(L)1 blockade plus chemotherapy in locally advanced oncogene-positive NSCLCs are limited. Nonetheless, two multicentre pooled studies have been focused on exploring the efficacy of neoadjuvant PD-1 blockade plus chemotherapy in oncogene-positive NSCLCs [15, 16]. In these two studies, inferior outcomes were achieved in the oncogene-positive cohorts compared with the oncogene-negative cohort with a pCR rate of 12.5% and an MPR rate of 37.5% [15], while neoadjuvant immunotherapy was found to yield the best outcomes among ICBs, TKIs, and chemotherapy in *EGFR*-mutant NSCLCs, with an MPR rate of 9% in the oncogene-positive NSCLC cohort [16] as presented in Table 4A. For *EGFR*-mutant patients, the efficacy of neoadjuvant PD-1 blockade plus chemotherapy seemed to be more sufficient in patients harbouring classical *EGFR* alterations including *exon19* deletions and *exon21 L858R* point mutations while not so satisfying in *exon17-25*-insertion cohorts [15, 16]. Moreover, two anecdotal reports recorded satisfying pathological outcomes of neoadjuvant PD-1 blockade plus chemotherapy regimen in both classical and rare *EGFR-*mutant NSCLCs; the two patients harboured *L858R* mutation [24], and *exon20 G779F* mutation [25], respectively, and they achieved pCR. Even though the above studies involved limited sample sizes, they still provided information supporting potential clinical feasibility of neoadjuvant PD-1 blockade-based immunotherapy in locally advanced *EGFR*-mutant NSCLCs. In our current study, in the oncogene-positive IO group, a pCR rate of 22.2% and an MPR rate of 44.4% were achieved, which were improved compared with the two multicentre studies above; and meanwhile, the oncogene alterations in the patients who had achieved pCR varied, including *EGFR exon19* deletions, *ERBB2* amplification, *KRAS G12V*, and *RET-KIF5B* fusion; the two patients harbouring *EGFR exon18/20* mutations achieved MPR or pCR, while the *L858R*-mutated patient hardly benefited from neoadjuvant PD-1 blockade plus chemotherapy. In addition, on the contrary, none of the patients who received neoadjuvant chemotherapy/TKIs had a pCR in our study, indicating the potential of neoadjuvant PD-(L)1 blockade-based treatment in patients with oncogene-positive NSCLC. Subgroup analysis of different mutation types in *EGFR*-mutated and other oncogene-altered NSCLCs with larger samples will further help screen the potential beneficial patients.

**Table 4:** Summaries of the recent studies focusing on neoadjuvant immunotherapy or targeted therapy in patients with potentially resectable, oncogene-positive NSCLCs

**(A) Summary of the two multi-centre study about PD-1 blockade plus chemotherapy in oncogene-mutant resectable NSCLCs**													
**Driver gene alterations**			**Number**		**ORR**		**pCR rates**		**MPR rates**			**Reference**	
*EGFR* 19del or 21L858R			15		10/15, 66.7%		2/15, 13.3%		8/15, 53.3%			Zhang et al. [16]	
			7		NA		0		0			Zhao et al. [15]	
*EGFR* 17–25 insertion			4		2/4, 50.0%		0		0			Zhang et al. [16]	
			4		NA		0		0			Zhao et al. [15]	
*BRAF* alteration			2		1/2, 50.0%		0		0			Zhang et al. [16]	
			1		NA		0		0			Zhao et al. [15]	
*HER2* insertion			3		2/3, 66.7%		1/3, 33.3%		1/3, 33.3%			Zhang et al. [16]	
*ALK* fusion			2		1/2, 50.0%		0		0			Zhang et al. [16]	
			3		NA		0		0			Zhao et al. [15]	
*RET* fusion			3		2/3, 66.7%		0		1/3, 33.3%			Zhang et al. [16]	
			1		NA		1/1, 100.0%		1/1, 100.0%			Zhao et al. [15]	
*ROS1* fusion			2		2/2, 100.0%		0		1/2, 50.0%			Zhang et al. [16]	
			2		NA		0		1/2, 50.0%			Zhao et al. [15]	
*MET* amplification			3		NA		0		0			Zhao et al. [15]	
**(B) Summary of trials of neoadjuvant TKIs of corresponding driver genes for resectable NSCLCs**													
**Study ID**	**Number**	**Oncogene**		**Regimen**		**ORR**		**R0 resection rates**		**pCR rates**	**MPR rates**		**Grade 3–4 TRAEs**
EMERGING-CTONG1103 [26]	72	*EGFR*		erlotinib, 42 days		54.1%		73.0%		0.0%	9.7%		0.0%
Xiong et al. [27]	19	*EGFR*		erlotinib, 56 days		42.0%		68.4%		0.0%	0.0%		10.5%
Zhang et al. [28]	35	*EGFR*		gefitinb, 42 days		55.0%		82.8%		12.1%	NA		0.0%
NEOS [29]	40	*EGFR*		osimertinib, 42 days		71.1%		94.0%		3.6%	10.7%		7.5%
Hu et al. [30]	13	*EGFR*		osimertinib, 75 days		69.0%		100.0%		0.0%	0.0%		0.0%
NAUTIKA1 (ALK^+^ cohort)	5	*ALK*		alectinib, 56 days		NA		100.0%		NA	NA		20.0%

Clinically, locally advanced NSCLCs are heterogeneous in terms of oncogene alterations, and multidimensional treatments are used for these patients. Some recent studies have focused on the neoadjuvant use of corresponding TKIs in *EGFR/ALK*-positive NSCLCs, and Table 4B shows the details of these trials. In *EGFR*-mutant patients, the pCR rate ranges from 0 to 12.1%, and the MPR rate varies between 0% and 10.7% [26–29]. Although neoadjuvant use of *EGFR*-TKIs has shown inferior clinical efficacy compared with first-line treatment for advanced NSCLCs [26] and is less effective than immunotherapy [16], it still provides alternative treatment options for *EGFR*-mutant resectable NSCLCs. In our study, none of the oncogene-positive patients who received neoadjuvant chemotherapy/TKIs achieved pCR; however, the MPR rate was 21.4%, and MPR was observed in one *ALK-EML4-*fused patient with TKI monotherapy (P19) and one *EGFR S768I* + *G719C* patient treated with chemotherapy plus TKI (P30). The ongoing Neo-ADAURA trial [31], the ALNEO trial [32] and the NAUTIKA1 trial are investigating the efficacy of the neoadjuvant use of various TKIs for corresponding driver genes in oncogene-mutant resectable NSCLCs. Moreover, the ongoing phase 2 Neo-DIANA study (NCT04512430) is evaluating the efficacy of the combination regimen of neoadjuvant PD-L1 blockade, atezolizumab, chemotherapy, and antiangiogenic agents in *EGFR*-mutant locally advanced NSCLC.

Regarding the efficacy of neoadjuvant PD-1 blockade plus chemotherapy in other oncogene-mutant NSCLCs, patients harbouring the *ROS1* fusion, *RET* fusion, or *HER2* insertion were found to be correlated with a better pathological response, while this combined regimen seemed to be less effective in patients with *MET*-amplified NSCLC [15, 16]. The results of our current study were in line with these findings. In the oncogene-positive IO group, the two patients harbouring the *ROS1-SDC4* fusion and *RET-KIF5B* fusion achieved a pCR and an MPR, respectively. Unfortunately, the patient with *MET* amplification suffered from disease progression after four cycles of neoadjuvant PD-1 blockade plus chemotherapy and had not undergone surgical resection. A recent study indicated that patients with *MET*-amplified NSCLCs with concurrent high PD-L1 expression had a decreased tumour response to anti-PD-1 immunotherapy [33], and neoadjuvant targeted therapy with MET-TKIs might be a better choice for locally advanced *MET*-amplified NSCLCs. Moreover, several resectable NSCLC patients harbouring a *ROS1* fusion or *RET* fusion have achieved satisfactory pathological outcomes after neoadjuvant therapy with corresponding TKIs [34–37], while whether neoadjuvant immunotherapy could benefit these patients should still be further verified.

Different histologic features in patients might also affect the tumour responses to neoadjuvant immunotherapy in locally advanced NSCLCs. In our study, although improvements in EFS and pCR/MPR with neoadjuvant immunotherapy were seen across all histologic features subgroups, the greater benefit seemed to be observed in squamous cell cancer, which was verified by partial immuno-therapy trials, including the AEGEAN study [38] and the NEOTORCH study [39]. The efficacy among oncogene-mutant NSCLCs with different histologic types should be further compared in prospective studies with larger participants.

The MPR has been applied as a major observational endpoint of the efficacy of PD-1 blockade-based neoadjuvant treatment in patients with NSCLC. Although PD-L1 expression was found to be positively correlated with the efficacy of immunotherapy in the first-line treatment of advanced oncogene-positive NSCLCs [40], no consensus has been reached regarding the clinical value of PD-L1 expression in determining the neoadjuvant treatment efficacy (pCR/MPR) or prognosis [16, 24]. In the phase 3 AEGEAN trial [38], PD-L1 status was a positive indicator of event-free survival, and in the CA209-159 trial [41], TMB was observed to be positively correlated with the efficacy of neoadjuvant PD-1 blockade-based treatment. Additionally, in a case report of *EGFR*-mutant NSCLC with a high PD-L1 TPS of 80% [24], pCR was achieved after neoadjuvant immunochemotherapy, indicating the promise of this combination regimen in *EGFR*-mutant NSCLCs, especially for those with concurrent high PD-L1 expression. In our current study, even though the sample size was limited, we still observed two patients with high PD-L1 expression who achieved pCR/MPR in the oncogene-positive IO group, and the pathological remission outcomes of the four TMB-H patients were pCR, one case of MPR, and one case of non-MPR. The associations between PD-L1 expression and the TMB and pathological response should be further explored in patients receiving neoadjuvant treatment for oncogene-mutant NSCLCs. The evaluations of tumour responses in patients harbouring specific oncogenic alterations and in patients with varied immunologic factors including PD-L1 expressions and tumour mutational burdens will be available in future well-designed prospective studies.

Although this study might shed light on the potential of neoadjuvant PD-1 blockade plus chemotherapy treatment for locally advanced oncogene-mutant NSCLCs, there are several limitations. The nonrandomized nature and limited sample size may have caused selection bias, including diversities in histologic subtypes and immunotherapy agents. Additionally, the sensitivities to immunotherapy may vary among the NSCLC patients harbouring different oncogene alterations. Well-designed prospective studies with larger sample sizes comparing the efficacy of neoadjuvant TKIs, ICB agents, and chemotherapy in resectable NSCLCs harbouring various oncogene alterations will ultimately bring out more accurate conclusions. Furthermore, in-depth studies on the underlying mechanisms will provide additional insights into this combination approach.

## Conclusion

Neoadjuvant PD-1 blockade plus chemotherapy could benefit patients with partially resectable, oncogene-positive NSCLC. In clinical practice, the detailed oncogene mutation types and immunotherapy response-related factors, including PD-L1 expression and the TMB, should be taken into consideration.

### Electronic supplementary material

Below is the link to the electronic supplementary material.

Supplementary Material 1

## Data Availability

All data supporting the findings of this study are available within the paper and its Supplementary Information.
