# Supplementary material for: Neoadjuvant PD-(L)1 blockade plus platinum-based chemotherapy for potentially resectable oncogene-positive non-small cell lung cancer
Source: World J Surg Oncol. 2024 Jun 18;22:159. doi: 10.1186/s12957-024-03434-1 (PMC11184808; doi:10.1186/s12957-024-03434-1)
Supplement: Supplementary file 1 — Supplementary Material 1 [file 12957_2024_3434_MOESM1_ESM.docx]

**Table S1**

Details of oncogene alterations, treatment regimens, tumour response and immunologic factors.

| ID | Demographics | Maximum tumour size (cm) | Oncogene alterations | Group | Treatment regimen | Radiographic response | | Pathological response | PD-L1 | TMB |
| --- | --- | --- | --- | --- | --- | --- | --- | --- | --- | --- |
| 01 | Female, 50 | 1.7 | EGFR exon19 del | Oncogene-positive IO | 1 cycle of PP+toripalimab, and 3 cycles of PP+tislelizumab | | PR | MPR | NA | NA |
| 02 | Female, 71 | 4.1 | EGFR L858R+G709A |  | 1 cycle of PP, and 2 cycles of PP+tislelizumab | | SD | non-MPR | TPS<1% | 19.54 muts/Mb |
| 03 | Male, 51 | 4.6 | EGFR G719C+E709V |  | 2 cycles of PP+ pembrolizumab | | PR | non-MPR | TPS 88% | 6.0 muts/Mb |
| 04 | Male, 59 | 6.8 | ERBB2 amplification |  | 3 cycles of TP+pembrolizumab | | PR | MPR | TPS 1% | 15.95 muts/Mb |
| 05 | Male, 64 | 7.0 | ERBB2 amplification |  | 4 cycles of TP+tislelizumab | | PR | non-MPR | TPS<1% | NA |
| 06 | Male, 75 | 3.1 | FGFR3-TACC3 fusion |  | 1 cycle of TP, and 2 cycles of TP+tislelizumab | | PR | pCR | TPS 20% | NA |
| 07 | Male, 67 | 5.1 | KRAS G12A |  | 2 cycles of PP, and 1 cycle of PP+tislelizumab | | SD | non-MPR | TPS<1% | NA |
| 08 | Male, 65 | 5.8 | KRAS G12A |  | 4 cycles of PP+pembrolizumab | | PR | pCR | TPS 15% | 13.98 muts/Mb |
| 09 | Male, 65 | 2.5 | KRAS G12C |  | 2 cycles of PP, and 2 cycles of PP+tislelizumab | | PR | pCR | TPS 75% | NA |
| 10 | Female, 54 | 4.6 | KRAS G12C |  | 1 cycle of PP, and 1 cycle of PP+sintilimab | | PR | non-MPR | TPS<1% | NA |
| 11 | Female, 70 | 2.3 | KRAS G12V |  | 1 cycle of PP, and 2 cycles of tislelizumab | | SD | MPR | TPS 30% | NA |
| 12 | Male, 67 | 4.7 | KRAS Q61H |  | 3 cycles of TP+toripalimab | | PR | non-MPR | TPS 20% | 3.0 muts/Mb |
| 13 | Male, 59 | 2.9 | KRAS Q61L |  | 1 cycle of PP, and 2 cycles of PP+tislelizumab | | SD | non-MPR | TPS 50% | NA |
| 14 | Female, 68 | 4.6 | MET exon14 skipping mutation |  | 3 cycles of PP, and 3 cycles of PP+camrelizumab | | PR | NA | TPS 70% | NA |
| 15 | Male, 73 | 4.6 | MET amplification |  | 4 cycles of TP+camreluzimab | | PD | NA | NA | 2.99 muts/Mb |
| 16 | Male, 54 | 4.2 | RET-KIF5B fusion |  | 2 cycles of PP+camrelizumab | | PR | MPR | TPS 10% | NA |
| 17 | Female, 68 | 3.4 | RET-KIF5B fusion |  | 3 cycles of TP+camrelizumab | | SD | non-MPR | TPS 90% | NA |
| 18 | Female, 45 | 5.7 | ROS1-SDC4 fusion |  | 4 cycles of PP+pembrolizumab | | PR | pCR | TPS 99% | 20.16 muts/Mb |
| 19 | Female, 69 | 2.7 | ALK-EML4 fusion | Oncogene-positive chemo/TKIs | 3 cycles of alectinib | | PR | MPR | TPS<1% | NA |
| 20 | Female, 57 | 2.4 | EGFR |  | 2 cycles of PP+endostar+gefitinib | | PR | non-MPR | TPS<1% | NA |
| 21 | Male, 61 | 2.6 | EGFR exon19 del |  | 1 cycle of PP, and 2 cycles of PP+osimertinib | | PR | non-MPR | TPS<1% | NA |
| 22 | Female, 59 | 3.1 | EGFR exon19 del |  | 2 cycles of PP+osimertinib | | PR | non-MPR | TPS 5% | NA |
| 23 | Female, 59 | 2.7 | EGFR exon19 del |  | 3 cycles of PP | | PR | non-MPR | NA | NA |
| 24 | Female, 33 | 4.3 | EGFR exon19 del |  | 3 cycles of PP | | SD | non-MPR | TPS<1% | NA |
| 25 | Female, 62 | 4.3 | EGFR L858R |  | 2 cycles of TP | | SD | non-MPR | NA | NA |
| 26 | Female, 52 | 2.0 | EGFR L858R |  | 2 cycles of PP, and 6 cycles of gefitinib | | SD | non-MPR | TPS<1% | NA |
| 27 | Female, 61 | 2.1 | EGFR |  | 3 cycles of PP | | PR | non-MPR | NA | NA |
| 28 | Female, 50 | 2.9 | EGFR |  | 2 cycles of PP | | PR | MPR | NA | NA |
| 29 | Male, 55 | 2.4 | EGFR S768I+G719C |  | 5 cycles of PP+afatinib | | PR | MPR | TPS<1% | NA |
| 30 | Female, 56 | 7.8 | HER2 exon20 insertion |  | 2 cycles of PP | | SD | non-MPR | TPS 1% | NA |
| 31 | Female, 64 | 2.7 | ROS1-SDC4 fusion |  | 2 cycles of PP | | SD | non-MPR | TPS 70% | NA |
| 32 | Male, 58 | 7.1 | - | Oncogene-negative IO | 2 cycles of TP+camrelizumab | | PR | non-MPR | TPS 30% | NA |
| 33 | Male, 54 | 4.5 | - |  | 2 cycles of TP+camrelizumab | | SD | MPR | TPS 10% | NA |
| 34 | Male, 59 | 7.1 | - |  | 3 cycles of TP+tislelizumab | | PR | MPR | TPS 70% | NA |
| 35 | Male, 67 | 4.0 | - |  | 4 cycles of TP+tislelizumab | | SD | MPR | NA | 16.9 muts/Mb |
| 36 | Male, 68 | 4.3 | - |  | 2 cycles of PP, and 3 cycles of PP+tislelizumab | | PR | pCR | NA | 2.84 muts/Mb |
| 37 | Male, 58 | 3.3 | - |  | 3 cycles of TP+pembrolizumab | | PR | pCR | TPS 15% | 7.27 muts/Mb |
| 38 | Male, 66 | 6.6 | - |  | 4 cycles of TP+pembrolizumab | | PR | MPR | TPS 85% | TMB-L |
| 39 | Male, 56 | 4.7 | - |  | 3 cycles of TP+toripalimab | | PR | pCR | TPS 10% | 5.98 muts/Mb |
| 40 | Male, 54 | 11.3 | - |  | 4 cycles of TP+toripalimab | | PR | non-MPR | TPS<1% | NA |
| 41 | Male, 63 | 6.2 | - |  | 3 cycles of TP+toripalimab | | PR | pCR | TPS>50% | NA |
| 42 | Male, 57 | 2.7 | - |  | 4 cycles of TP+toripalimab | | PR | pCR | TPS<1% | NA |
| 43 | Male, 63 | 2.9 | - |  | 2 cycles of TP+toripalimab | | PR | pCR | TPS 40% | NA |
| 44 | Female, 63 | 5.1 | - |  | 3 cycles of TP+toripalimab | | PR | non-MPR | TPS 2% | NA |
| 45 | Male, 65 | 4.1 | - |  | 4 cycles of TP+sintilimab | | PR | MPR | NA | 11.25 muts/Mb |
| 46 | Male, 65 | 4.5 | - |  | 2 cycles of TP+durvalumab | | SD | pCR | TPS 70% | NA |
